# Limited precut sphincterotomy combined with endoscopic papillary balloon dilation for common bile duct stone removal in patients with difficult biliary cannulation

**DOI:** 10.1186/s12876-016-0486-4

**Published:** 2016-07-12

**Authors:** Chung-Mou Kuo, Yi-Chun Chiu, Chih-Ming Liang, Lung-Sheng Lu, Wei-Chen Tai, Yuan-Hung Kuo, Cheng-Kun Wu, Seng-Kee Chuah, Chi-Sin Changchien, Chung-Huang Kuo

**Affiliations:** Department of Internal Medicine, Division of Hepato-Gastroenterology, Kaohsiung Chang Gung Memorial Hospital, 123 Ta Pei Road, Niao-Sung Dist. 833, Kaohsiung City, Taiwan; Chang Gung University College of Medicine, Kaohsiung, Taiwan

**Keywords:** Precut sphincterotomy, Endoscopic sphincterotomy, Endoscopic papillary balloon dilation, Difficult biliary cannulation, Common bile duct stones

## Abstract

**Background:**

Difficult biliary cannulation in endoscopic retrograde cholangiopancreatography (ERCP) can result in failure of common bile duct (CBD) stone removal and pancreatitis. The present study aimed to report the efficacy and safety of limited precut sphincterotomy (PS) combined with endoscopic papillary balloon dilation (EPBD) for CBD stone removal in patients with difficult biliary cannulation, and the complications associated with this combined procedure.

**Methods:**

A total of 3305 patients underwent ERCP in our hospital between October 2009 and September 2014 and 258 were diagnosed with difficult biliary cannulation. Of these 258 patients, 58 underwent limited PS combined with EPBD for CBD stone removal, and these 58 patients were included in this retrospective study.

**Results:**

The overall success rate was 94.8 % (55/58), and the success rate for single-session removal was 87.9 % (51/58). The mean procedure time was 41 ± 11.48 min (range, 20–72 min). Mechanical lithotripsy was needed in 10.3 % (6/58) of patients. Procedure-related complications included bleeding in 3.4 % (2/58), pancreatitis in 8.6 % (5/58) and biliary tract infection (BTI) in 1.7 % (1/58) of patients.

**Conclusions:**

The therapeutic outcome of limited PS combined with EPBD for CBD stone removal in patients with difficult biliary cannulation was good with an acceptable complication rate. It could be an alternative to PS and “early” limited PS should be used for prompt identification of the bile duct. Limited PS combined with EPBD is safe and effective for CBD stone removal in patients with difficult biliary cannulation.

## Background

A common bile duct (CBD) stone complicated with cholangitis, obstructive jaundice, or pancreatitis is a common disease of the biliary tract. Gaining access to the CBD is the most importance step for successful therapeutic endoscopic retrograde cholangiopancreatography (ERCP) [1–7]. The cannulation success rate depends on patient selection, the utilization of a specialized catheter, and the skill and experience of the endoscopist [2, 7]. The overall success rate of cannulation has been reported to be 90–95 % even when performed by experts [1–5]. However, in 5–10 % of cases, the CBD remains inaccessible, necessitating precut sphincterotomy (PS) or fistulotomy (PF), percutaneous transhepatic biliary drainage (PTBD), endoscopic ultrasound (EUS)-guided drainage, or surgery [1–7]. Difficult biliary cannulation is defined as a situation in which an endoscopist, using the regular cannulation technique, fails to cannulate the bile duct within a certain amount of time or after a certain number of attempts [3, 6]. Some investigators have proposed the definition of difficult biliary cannulation as (1) failed cannulation within 10 min, (2) >5 pancreatic cannulation attempts, or (3) 5–10 attempts at the papilla without a time limit [1–7]. Difficult biliary cannulation leads to prolonged papillary manipulation resulting in not only tissue edema but also repeated attempts at cannulation or contrast injection of the pancreatic duct, and these factors have been reported to cause post-ERCP pancreatitis in 4.3–11.3 % of cases [1, 3, 5, 6]. Needle-knife PS is the most commonly used procedures in patients with difficult biliary cannulation, and it has been reported to have success rates of 74.5–98.2 % [1–4, 6, 7]. However, PS is associated with post-ERCP complications such as acute pancreatitis, duodenal bleeding and perforation, and is often regarded as a risky procedure, with complication rates of 2–34 % [1–7]. Some published studies have reported that sequential endoscopic papillary balloon dilation (EPBD) after endoscopic sphincterotomy (EST) is safe and effective for the management of CBD stones and could decrease the occurrence of complications, including procedure-related pancreatitis [8–13]. However, reports on the efficacy of limited PS combined with EPBD for CBD stone removal in patients with of difficult biliary cannulation are scarce.

Therefore, the present study aimed to report the efficacy and safety of limited PS combined with EPBD for CBD stone removal in patients with difficult biliary cannulation, and the complications associated with this combined procedure.

## Methods

### Patients

A total of 3305 patients underwent ERCP in our hospital between October 2009 and September 2014 and 258 were diagnosed with difficult biliary cannulation. Of these 258 patients, 58 of them had their index ERCP with successful cannulations and underwent subsequent limited PS combined with EPBD for CBD stone removal. These 58 patients were analyzed in this retrospective study. On the other hand, two hundred of these patients encountered a first unsuccessful ERCP due to difficult biliary cannulation and then decided not to have a second one (none of them had received limited precut procedure). One hundred and fifty-five of them chose to receive surgery; 42 of them were treated by percutaneous biliary drainage and 3 received supportive treatments.

**Fig. 1:**
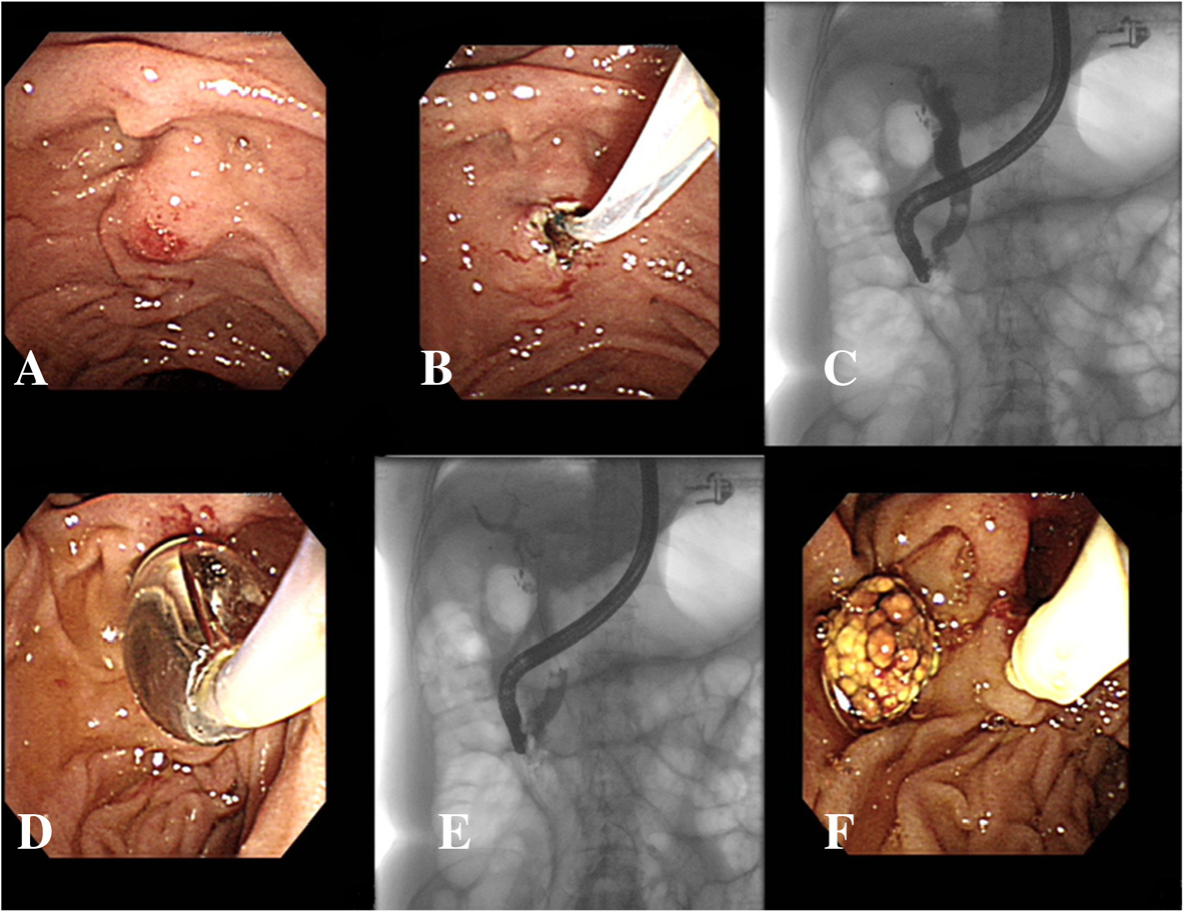
**a** Difficult biliary cannulation was due to failure of 10 attempts at duodenal papilla; (**b**) Limited precut sphincterotomy was performed with the extent of cutting was less than half the length of the papillary mound; (**c**) Common bile duct stone was found after successful biliary cannulation; (**d**) Endoscopic papillary balloon dilation was performed after limited precut sphincterotomy; (**e** and **f**) Common bile duct stone was extracted by retrieval balloon

The definition of difficult biliary cannulation in our study was as follows: (1) failed cannulation within 10 min (2) 5 passages or injections of the pancreatic duct, or (3) 10 attempts at the papilla without a time limit (Fig. 1a). We stopped anticoagulant administration such as aspirin for 7 days before the procedures in those who were prescribed for primary prevention. For those who received anticoagulant for secondary prevention in low cardiovascular risk patients, we stopped clopidogrel, prasugrel, ticagrelor and coumadin 5 days before ERCP according to British Society of Gastroenterology and European Society of Gastrointestinal endoscopy [14, 15]. For patients with high cardiovascular risks, the procedures were postponed if possible until anticoagulant could be discontinued safely (usually >12 months after insertion of drug-eluting coronary stents or >1 month after insertion of bare metal coronary stents). However, when an emergent or semi-emergent indication like an impacted stone or jaundice in need of immediate action was encountered, cardiologists were routinely consulted and replaced by other emergent non-endoscopic bilary driange procedures. Prophylactic NSAIDs were given to all patients to reduce risk of post-ERCP pancreatitis routinely in our department.

The patients received pharyngeal anesthesia with xylocaine spray in the same manner as that for general endoscopy. Hyoscine-N-butylbromide (20 mg) was administered intramuscularly before the procedure, and meperidine (30 to 50 mg) was administered before EPBD. ERCP was performed using a side-view endoscope (JF 260v and TJF 240; Olympus, Tokyo, Japan) after selective cannulation of the CBD with a cholangiography catheter (PR-113Q, Olympus). We preferred a needle-knife sphincterotome (KD-V441, Olympus) in all cases. Two highly experienced endoscopists [the first and second authors], with experiences of more than 3000 ERCPs procedures each, and ongoing workloads of more than 250 ERCPs procedures each annually, performed all the limited PS combined with EPBD procedures. Diathermy was applied with a blended current (20 W cut and 20 W coagulation) using the ESG 100 system (Olympus). The incision started from the lip of the papillary orifice (at the 11-o’clock position) and proceeded upward over the papillary mound. The extent of cutting in limited PS is less than half the length of the papillary mound (Fig. 1b). To perform EPBD after cannulation of the bile duct with limited PS (Fig. 1c), a 0.035-inch guide-wire (Zebra Exchange Guide-wire; Microvasive Boston Scientific, Watertown, MA) was inserted into the bile duct through the catheter. After the guide-wire was inserted deeply into the bile duct, the catheter was removed with the guide-wire left in place. A balloon-tipped catheter (5.5 cm long and 8–20 mm wide; Microvasive Boston Scientific), was inserted over the guide-wire so that the balloon was extended across the papilla. The balloon was inflated to 8–20 mm in diameter with saline solution to dilate the papilla at progressively increasing pressures of 3 to 8 atm for 2 min, according to the size of the CBD stones (Fig. 1d). After removing the dilation catheter, stones were extracted with a basket catheter or retrieval balloon (Fig. 1e, f). Endoscopic mechanical lithotripsy (EML) was used to crush stones >15 mm in diameter when extraction of these stones was difficult after EPBD. When stones were not extracted completely, a biliary stent was inserted and the residual stones were removed after 3–7 days without repeating EPBD. A prophylactic pancreatic stent was not used after EPBD. Complete stone removal was defined as the absence of bile duct stones on a balloon occlusion cholangiogram.

### Definitions

A procedure-related complication was defined as any adverse event related to the procedure, including pancreatitis, duodenal bleeding, perforation and biliary tract infection. Acute pancreatitis was defined as abdominal pain occurring within 24 h after the procedure in association with high serum amylase and lipase levels equivalent to at least 3 times the normal ranges and basal levels on the day after ERCP [2]. Bleeding was defined as any drop of over 15 % in the hemoglobin level, any clinical sign of gastrointestinal bleeding (e.g., hematemesis and tarry stool), or the need for blood transfusion [2]. Perforation was defined as the leakage of contrast medium into the retroperitoneum or intraabdominal cavity during ERCP or evidence of retroperitoneal-free air on abdominal plain radiography or computer tomography (CT) [2]. Biliary tract infection was defined as presence of fever and/or chills, abdominal pain, jaundice, and leukocytosis.

## Results

This study included 58 patients (28 men and 30 women) with CBD stones who underwent limited PS combined with EPBD. The mean age of the patients was 64.02 years (range, 26–96 years). The characteristics of the patients are presented in Table 1. The procedure findings during limited PS combined with EPBD are presented in Table 2. Complete removal of CBD stones was achieved in 55 patients (94.8 %). Of these 55 patients, 51 patients (87.9 %) required 1 session and 4 patients required 2 sessions for complete removal (Table 2). The mean size of the CBD stones was 1.11 ± 0.40 cm (range, 0.4–2.0 cm) and the mean diameter of CBD was 1.47 ± 0.44 cm (range, 0.7–2.6 cm). Of the 58 patients, 28 patients had one stone, 14 patients had 2 stones, and 16 patients had ≥3 stones. Additionally, among the 58 patients, 41 patients had distal CBD narrowing, 41 patients had jaundice, 28 patients had biliary tract infection (BTI), 19 patients had duodenal periampullary diverticulum, and 13 patients had impacted CBD stones. Removal was successful in 100 % (19/19) of patients with stones ≤1 cm and 92.3 % (36/39) of patients with stones >1 cm (*p* = 0.544, Fisher’s exact test). EML was used to crush stones >15 mm in diameter when extraction of these stones was difficult after EPBD in 6 patients (10.3 %). Removal failed in 3 patients because of large stones (1.5–2.0 cm) and CBD segmental strictures, and all the 3 patients underwent surgery for removal of the CBD stones. Among the 58 patients, 2 patients (3.4 %) had procedure-related duodenal bleeding and were successfully treated with endoscopic epinephrine injection. None of the patients with CAD, heart disease or stroke requiring anticoagulant treatment; cirrhosis; or end-stage renal disease (ESRD) had procedure-related duodenal bleeding or perforation. Among the 58 patients, 5 patients (8.6 %) had procedure-related acute pancreatitis (mild pancreatitis). Of the 5 patients, 2 were men (7.1 % of the 28 men) and 3 were women (10 % of the 30 women), and 2 of the 5 patients were under 60 years of age. One patient (1.7 %) had procedure-related BTI, and the pathogen was *Escherichia coli*. The overall complication rate was 13.7 % (8/58). The mean follow-up period was 29.0 ± 14.9 months (range, 1–60 months), and no recurrence of symptomatic CBD stones was noted during the follow-up period. The mean procedure time of limited PS combined with EPBD was 41.0 ± 11.5 min (range, 20–72 min).

**Table 1:** The characteristics of 58 patients underwent limited PS combined with EPBD

Characteristics	Patient number
Gender (M:F)	28:30
Mean age (range) yr	64.02 ± 16.37 (26–96)
Age (<60 : ≥60 : ≥70) yr	18:40:23
Gallstone	31
Prior cholecystectomy	14
Acute pancreatitis	8
Jaundice	41
Biliary tract infection	28
Liver cirrhosis	7
Hypertension	22
Diabetes mellitus	11
ESRD	4
CAD and heart disease	8
Hyperlipidemia	15
Malignancy	6
Stroke	3
COPD and asthma	4

*PS* precut sphincterotomy, *EPBD* endoscopic papillary balloon dilation, *CAD* coronary artery disease, *COPD* chronic obstructive pulmonary disease, *ESRD* end-stage renal disease

**Table 2:** Procedure findings during limited PS combined with EPBD

Procedure findings	Number or size
Complete bile duct stone clearance	55
Number of sessions required to complete bile duct stone clearance (1:2)	51:4
Successful removal of CBD stone (≤1 cm : >1 cm)	19:36
Mean stone size (range)	1.11 ± 0.40 (0.4–2.0) cm
Stones size (≤1 cm : >1 cm)	19:39
Stone number (1:2: ≥3)	28:14:16
Mean CBD diameter (range)	1.47 ± 0.44 (0.7–2.6) cm
CBD diameter (≤0.8 cm : >0.8 cm)	4:54
Periampullary diverticulum	19
Distal CBD narrowing	41
Impacted CBD stone	13
Mechanical lithotripsy	6
Procedure time	41 ± 11.48 (20–72) min

*PS* precut sphincterotomy, *EPBD* endoscopic papillary balloon dilation, *CBD* common bile duct

## Discussion

Current study achieved a high success rate in CBD stones removal of 94.8 % with a relative shorter mean procedure time needed was 41.0 ± 11.5 min (range, 20–72 min) in patients who underwent limited PS combined with EPBD. Difficult biliary cannulation is one major reason that influences the success rates and procedural times used to remove bile duct stones during ERCP. It is usually decided depending on the length of time, number of selective biliary cannulation attempts, or the number of unwanted pancreatic cannulations, or insertion of a guide-wire into the pancreatic duct [2]. In approximately 5–10 % of patients, biliary cannulation cannot be achieved, and further complex techniques are needed. Prolonged and repeated attempts of biliary cannulation (more than 1 cannulation attempt or the cannulation time was greater than 10 min) resulted in extensive injury to the papilla and lead to post-ERCP pancreatitis [16, 17].

The causes of difficult biliary cannulation are related to anatomical and physiological factors, such as a floppy papilla, small papillary orifice, cervical of the papilla, periampullary diverticulum, and surgically altered anatomy, and improper positioning of the duodenoscope [3, 4, 18]. Additionally, pathological conditions, such as stenosis of Oddi’s sphincter, duodenal inflammation, ampullary and papillary neoplasms, large size or number of stones, impacted stones, bile duct strictures, and a relatively narrow distal CBD compared with the stone size can cause difficult biliary cannulation [3, 4, 19]. Distal CBD narrowing (41/58) was the major cause of difficult biliary cannulation in our study, and it may be related to chronic cholangitis. A duodenal periampullary diverticulum (19/58) and impacted CBD stone (13/58) were also common causes of difficult biliary cannulation in our study.

The solutions for overcoming difficult biliary cannulation in order to increase the success rate in CBD stones removal and shortening the procedure time include changing the catheter or operator, or applying a more aggressive method, keeping in mind the increased risk of complications [6]. The more aggressive methods include needle-knife PS, papillary roof excision, transpancreatic sphincterotomy, transpancreatic stenting, the double wire technique, persistence, papillectomy, and the use of a special knife [6]. If endoscopic methods fail, the transhepatic route can be used directly without an endoscopist or the rendezvous technique can be applied, depending on the cause of difficult biliary cannulation [6]. Needle-knife PS is the most commonly used technique for difficult biliary cannulation, and it has a success rate of 74.5–98.2 % and complication rate has been reported to be 2–34 % such as bleeding (2–9.5 %), pancreatitis (0.5–7.6 %) and perforation (1.4–3 %) [1–4, 6, 7, 18, 20]. Some studies have recommended the used of needle-knife PS in the following situations: (1) stone impacting the papillary orifice, (2) significant eminence of the ampulla or dilation at the end of the CBD, (3) acute obstructive suppurative cholangitis and pancreatitis due to biliary disorder, and (4) Billroth II gastrectomy [7]. However, needle-knife PS is contraindicated for a small flat papilla, periampullary diverticulum and malignant change of the papilla, because the procedure can potentially make the cannulation approach difficult or unsafe to perform [7]. The early application of needle-knife PS for difficult biliary cannulation has been reported to be time-saving, safe, and effective, with no increase in the complication rate [1, 2, 4, 7]. Limited endoscopic sphincterotomy (EST) could minimize the risk of complications that occur after complete EST such as bleeding, bile reflux and biliary tract malignancy [8]. Among the 55 patients with successful CBD stone removal, the success rate for single-session removal was 87.9 % (51/58) while the other four needed more than once subsequent session removal of stones but were all removed eventually(94.8 % overall). The therapeutic outcome in the present study was as good as that reported previously for patients without difficult biliary cannulation who underwent EST or EPBD (79–100 %), or combination therapy (80–100 %) with an acceptable complication rate (13.7 %) [9–13, 18–25] with only mild procedure related pancreatitis (8.6 %) and bleeding (3.4 %).

In the present study, no difference was noted in the removal rate between CBD stones ≤1 cm (100 %, 19/19) and those >1 cm (92.3 %, 36/39) (*p* = 0.554, Fisher’s exact test). The proportion of patients who need EML to crush stones when extraction of these stones was difficult after EPBD was 10.3 % in the present study. This did not differ from the proportion of patients without difficult biliary cannulation who underwent EST and EPBD combination therapy and needed EML in previous studies (0–33 %) [8–12, 23]. The procedure time for CBD stone removal was longer in the present study (mean, 41.0 ± 11.5 min; range, 20–72 min) than in previous studies that reported patients without difficult biliary cannulation (EST: mean, 21.9 ± 14.7 min; range, 3–63 min and EST combined with EPBD: mean, 13.1 ± 6.6 min; range, 4–35 min) [12]. The reason for the longer procedure time in the present study might be the extra time required owing to difficult biliary cannulation. There was no definite recurrence of symptomatic CBD stones during the follow-up period (mean, 29.0 ± 14.9 months; range, 1–60 months) in our study.

The present study had some limitations. First, this was a non-randomized retrospective study. Second, the sample size was small for statistical analysis such as univariate and multivariate analysis to evaluate clinical factors associated to the outcome. A larger sample size is needed to further confirm the results of the present study.

## Conclusions

The therapeutic outcome of limited PS combined with EPBD for CBD stone removal in patients with difficult biliary cannulation was good with an acceptable complication rate. It could be an alternative to PS and “early” limited PS should be used for prompt identification of the bile duct. Limited PS combined with EPBD is safe and effective for CBD stone removal in patients with difficult biliary cannulation.

## Abbreviations

CBD, common bile duct; CT, computer tomography; EML, endoscopic mechanical lithotripsy; EPBD, endoscopic papillary balloon dilation; ERBD, endoscopic papillary balloon dilation; ERCP, endoscopic retrograde cholangiopancreatography; ESRD, end-stage renal disease; EST, endoscopic sphincterotomy; EUS, endoscopic ultrasound; PF, precut fistulotomy; PS, precut sphincterotomy; PTBD, percutaneous transhepatic biliary drainage
